# An NMR Metabolomics Analysis Pipeline for Human Neutrophil Samples with Limited Source Material

**DOI:** 10.3390/metabo15090612

**Published:** 2025-09-15

**Authors:** Grace Filbertine, Genna A. Abdullah, Lucy Gill, Rudi Grosman, Marie M. Phelan, Direkrit Chiewchengchol, Nattiya Hirankarn, Helen L. Wright

**Affiliations:** 1 Institute of Life Course and Medical Sciences, University of Liverpool, Liverpool L7 8TX, UK; grace.filbertine@liverpool.ac.uk (G.F.); g.abdullah@liverpool.ac.uk (G.A.A.);; 2 High-Field NMR Centre, University of Liverpool, Liverpool L69 7ZB, UK; 3 Institute of Systems, Molecular and Integrative Biology, University of Liverpool, Liverpool L69 7ZB, UK; 4 Faculty of Medicine, Chulalongkorn University, Bangkok 10330, Thailand

**Keywords:** NMR, metabolomics, neutrophils, paediatric medicine

## Abstract

**Background/Objectives:** Untargeted ^1^H NMR metabolomics is a robust and reproducible approach used to study the metabolism in biological samples, providing unprecedented insight into altered cellular processes associated with human diseases. Metabolomics is increasingly used alongside other techniques to detect an instantaneous altered cellular function, for example, the role of neutrophils in the inflammatory response. However, in some clinical settings, blood samples may be limited, restricting the amount of cellular material available for a metabolomic analysis. In this study, we wanted to establish an optimal 1D ^1^H NMR metabolomic pipeline for use with human neutrophil samples with low amounts of input material. **Methods**: We compared the effect of different neutrophil isolation protocols on metabolite profiles. We also compared the effect of the absolute cell counts (100,000 to 5,000,000) on the identities of metabolites that were detected with an increasing number of scans (NS) from 256 to 2048. **Results/Conclusions**: The variance in the neutrophil profile was equivalent between the isolation methods, and the choice of isolation method did not significantly alter the metabolite profile. The minimum number of cells required for the detection of neutrophil metabolites was 400,000 at an NS of 256 for the spectra acquired with a cryoprobe (700 MHz). Increasing the NS to 2048 increased metabolite detection at the very lowest cell counts (<400,000 neutrophils); however, this was associated with a significant increase in the analysis time, which would be rate-limiting for large studies. The application of a correlation-reliability-score-filtering method to the spectral bins preserved the essential discriminatory features of the PLS-DA models whilst improving the dataset robustness and analytical precision.

## 1. Introduction

The use of untargeted metabolite profiling through NMR provides a cost-effective approach for capturing a broader metabolite fingerprint, offering a robust replicability and an unbiased approach for identifying novel biomarkers in complex biological samples. Untargeted NMR-based metabolomics is being increasingly applied to identify novel biomarkers associated with diseases, progression stages, and treatment responses, providing crucial insights to enhance early diagnostics and personalised therapeutic strategies [1]. While the human genome has been fully sequenced, with nearly 21,000 protein-coding genes discovered, human metabolomics remains a rapidly growing field [2,3]. To date, around 114,100 metabolites have been identified, with detailed information on their biological functions, physiological concentrations, disease associations, chemical interactions, metabolic pathways, and reference spectra compiled in the Human Metabolome database (https://hmdb.ca) since its inception in 2007 [4]. Integrating metabolomics with genotyping, as well as proteomic and transcriptomic data, holds promise in advancing biomarker discovery for complex diseases and tailoring preventive strategies. A metabolomic analysis of patient-derived samples, such as blood, urine, saliva, or tears, offers valuable insights into disease mechanisms, diagnoses, and progression [5,6,7,8]. By capturing distinct metabolic signatures associated with specific conditions, metabolomics can facilitate an early, minimally invasive diagnosis. For instance, profiling the metabolites in urine and cells has successfully distinguished between cancerous and healthy samples, showcasing the potential of metabolomics in identifying novel biomarkers. These biomarkers support a precision medicine approach, offering the ability to design tailored interventions and develop alternative, less invasive diagnostic methods, ultimately enhancing the predictive capabilities for prognoses and disease progression.

Neutrophils are innate immune cells that play an important role in the host defence against infection through the phagocytosis of microorganisms and the production of inflammatory cytokines and chemokines [9]. They are also implicated in the pathogenesis of a number of immune-mediated inflammatory diseases, such as rheumatoid arthritis (RA) and adult and juvenile systemic lupus erythematosus (SLE), due to their ability to secrete tissue-damaging and pro-inflammatory molecules, including reactive oxygen species (ROS), neutrophil extracellular traps (NETs), and proteases such as collagenase and gelatinase [10]. Neutrophils are terminally differentiated leukocytes with a very short half-life of under 24 h. As such, the metabolic signatures from neutrophils are frequently lost from the analysis of whole-blood samples. In addition, the isolation of peripheral blood monocytes (PBMCs) using media such as lymphoprep discards the neutrophil population, meaning that neutrophils are often overlooked in studies of human diseases. Despite extensive advancements in human metabolomic research, the neutrophil metabolome remains a largely unexplored area. The latest report from the Consortium of Metabolomics Studies (COMETS), which spans 47 prospective cohort studies worldwide and features significant data from blood samples collected between 1985 and 2017, has enhanced the understanding of metabolomics in various contexts [11]. However, none of these studies have specifically addressed neutrophil-targeted metabolomics in human samples. Recent NMR studies by Richer et al. and Chokesuwattanaskul et al. [12,13] developed protocols to study neutrophil metabolomics, focusing on intracellular metabolites in healthy adult neutrophils. These studies have laid the groundwork for translational neutrophil metabolomic research using clinical samples from adults and children with inflammatory and metabolic diseases.

One drawback of the recently published protocols for human neutrophils is the amount of material required to measure the neutrophil metabolites. The Richer et al. study extracted intracellular metabolites from 20 million neutrophils using a chloroform-based extraction protocol [12]. Meanwhile, Chokesuwattanaskul et al. [13] optimised a protocol for a minimum cell count of 5 million neutrophils and a number of scans (NS) of 512 by introducing a heat-shock step to preserve the metabolites and reduce the number of washing steps. Additionally, the use of HEPES-free media was employed to enhance the low signal to noise quality in the spectra, hence achieving the optimal conditions for metabolite detection. This established protocol was then used for a recent study that investigated an altered neutrophil metabolism in people with RA [14]. However, in cases where clinical sample collection is restricted—for example, by ethics in the case of paediatric samples, or by clinical neutropenia in diseases such as systemic lupus erythematosus—it may not be possible to isolate 5 million neutrophils for an NMR metabolomic analysis. We therefore wanted to optimise the NMR metabolomic protocols for use in clinical studies with limited material for metabolite extraction. The aim of this study was to determine the minimal cell count required for an NMR metabolomic analysis of human neutrophils, by optimising the neutrophil isolation protocol and the NMR analysis parameters to maximise the spectral analysis of neutrophil metabolites.

## 2. Materials and Methods

### 2.1. Ethical Approval

This study was approved by the University of Liverpool Central University Research Ethics Committee (No. 10956). All the study participants provided written informed consent in accordance with the Declaration of Helsinki. All the study participants were healthy, free from infection, and over the age of 18 years.

### 2.2. Neutrophil Isolation

Human peripheral blood samples were obtained by venipuncture and transferred into lithium-heparin vacutainers. The neutrophils were isolated immediately in order to maintain their viability. Two different neutrophil isolation protocols were compared. For the first method, whole blood was mixed with HetaSep (STEMCELL Technologies, Cambridge, UK) at a dilution ratio of 5:1 and incubated at 37 °C for 30 min to allow for the sedimentation of the erythrocytes. The nucleated cells were then layered onto a Ficoll-Paque Plus solution (Merck, Gillingham, UK) in a 1:1 ratio and centrifuged for 30 min at 500 g. Contaminated erythrocytes were removed with a hypotonic ammonium chloride lysis buffer and the cells were resuspended in RPMI-1640 media without the supplementation of phenol red buffer or HEPES. The cellular viability was assessed with the trypan blue (Merck, Gillingham, UK) exclusion method in a Neubauer haemocytometer. The neutrophil purity was confirmed with Wright Giemsa staining and morphology. Only samples with ≥95% viability and purity were eligible for further experiments. In the second method, ultrapure neutrophils (>99.9% purity) were obtained using a negative selection kit for neutrophil isolation, employing magnetic bead separation (STEMCELL Technologies, Cambridge, UK). For this method, whole blood was mixed with HetaSep (STEMCELL Technologies, Cambridge, UK) at a dilution ratio of 5:1 and incubated at 37 °C for 30 min to allow for the sedimentation of the erythrocytes. The nucleated cells were then mixed with an equal volume of ice-cold isolation buffer (PBS, 2% BSA, 100 μL 0.2 M EDTA). The suspension was centrifuged at 400× *g* for 5 min, and the pellet was resuspended in 2 mL of isolation buffer. An antibody cocktail (25 μL) containing tetrameric antibody complexes that recognised cell markers of unwanted leukocytes and erythrocytes and magnetic particles was added to the suspension, and the mixture was incubated on ice for 5 min. Following this, 50 μL of a magnetic bead solution was added for 5 min before placing the mixture in a magnetic chamber for 3 min. The unlabelled neutrophils were decanted into a clean tube and resuspended in HEPES-free and phenol red-free RPMI 1640 media. The cellular viability was assessed with the trypan blue (Merck, Gillingham, UK) exclusion method in a Neubauer haemocytometer. The neutrophil purity was confirmed at >99% with Wright Giemsa staining and morphology.

### 2.3. Preparation of Samples for NMR Metabolomics

The neutrophils were processed using our previously optimised protocol for neutrophil metabolomics, where we established the need for a heat-shock step to preserve intracellular metabolites [13]. The neutrophils were pelleted by centrifugation at 1000× *g* at 25 °C for 2 min and the supernatant was aspirated. The cell pellets were heated at 100 °C for 1 min prior to being snap-frozen using liquid nitrogen and stored at −80 °C for further analysis.

### 2.4. Metabolite Extraction

The neutrophil metabolites were extracted using an established method optimised for human neutrophils [13,15]. A mixture of 50:50 *v*/*v* ice-cold HPLC-grade acetonitrile/water (ddH_2_O) was added to each sample (500 μL per cell pellet), followed by a 10 min incubation on ice. The samples were sonicated three times for 30 s at 23 kHz and an amplitude of 10 μm using an exponential probe with 30 s of rest in between each sonication in an ice water bath. The sonicated samples were centrifuged at 12,000× *g* for 5 min at 4 °C, followed by the transfer of the supernatants to cryovials and flash-freezing in liquid nitrogen prior to lyophilisation. All the lyophilised samples were stored at −80 °C prior to their analysis in a spectrometer.

### 2.5. NMR Spectral Acquisition and Quality Assurance of Acquired Spectra

The lyophilised metabolite pellets were resuspended in 200 μL of 100 mM deuterated sodium phosphate buffer, with a pH of 7.4, along with 100 μM of TSP-d4 and 0.05% NaN_3_. All the samples were vortexed for 20 s and centrifuged at 12,000× *g* for 1 min at 20 °C prior to transferring them into 3 mm (outer diameter) NMR tubes for acquisition using a 700 MHz Avance IIIHD Bruker NMR spectrometer equipped with a TCI cryoprobe and a chilled SampleJetTM autosampler. A spectrometer quality assurance was completed daily via temperature calibration to 25 °C (with a margin of error of 0.1 °C) by a methanol thermometer (cat. number Z10627; 99.8% methanol-d4, 5 mm, Bruker, UK) [16] and 3D shimming on Bruker standard 2 mM sucrose (cat. number Z10902; 2 mM sucrose, 0.5 mM DSS, and 2 mM NaN_3_ in H_2_O/D_2_O, 90/10, 5 mm Bruker, UK) to ensure that the line width at half the height of the DSS reference peak was within the acceptance criteria (<1 Hz). The spectra were acquired using standard (vendor-supplied) 1D ^1^H Carr–Purcell–Meiboom–Gill (CPMG; cpmgpr1d) spectra with 256, 512, 1024, or 2048 transients as specified, a 15 ppm spectral width, 32K points, a 9.6 ms echo time, a 3.1 s acquisition time, and a 4 s interscan delay. This approach prevented interference from macromolecules, which may have been present after the extraction (Supplementary Figure S1). Additionally, 1D ^1^H nuclear Overhauser effect spectroscopy (NOESY) pre-saturation experiments (noesypr1d) were performed to verify the shimming quality and ensure effective water suppression for each sample during data acquisition. This step was essential to maintain the spectral clarity and consistency across the samples. All the acquired spectra were automatically phase-referenced to 3-(trimethylsilyl) propionic-2,2,3,3-d4 acid sodium salt (deuterated trimethylsilyl propionate; TSP-d4) at 0 ppm and phasing- and baseline-corrected using the automated macro (apk0.noe) provided within TopSpin v4.4.1 (Bruker Corporation), followed by a compliance check according to the minimum quality criteria outlined by the Metabolomics Standards Initiative (MSI) to suppress the macromolecule signals, selectively accentuate small-molecule metabolite signals, and ensure consistent line widths, baseline corrections, and water suppression. All the spectra were thoroughly evaluated to ensure that they met the current best practice of the Metabolomics Society [15,17,18,19], ensuring a robust consistency in phasing, baseline correction, peak alignment, chemical shift reference to TSP-d4 at 0 ppm, line width, and water suppression conditions for neutrophil intracellular metabolomic reporting. Only the technical replicates with the lowest line width at the half-height from each sample that passed the quality control process, including a flat baseline, consistent line widths, and water suppression consistency, were selected. Any spectra that did not meet the requirements of the recommended reporting standards were subsequently removed from further processing [17]. The samples that failed QC were stored at 4 °C for up to 14 days to allow for a re-analysis. Samples that failed QC after three attempts were excluded from the analysis.

**Figure 1:**
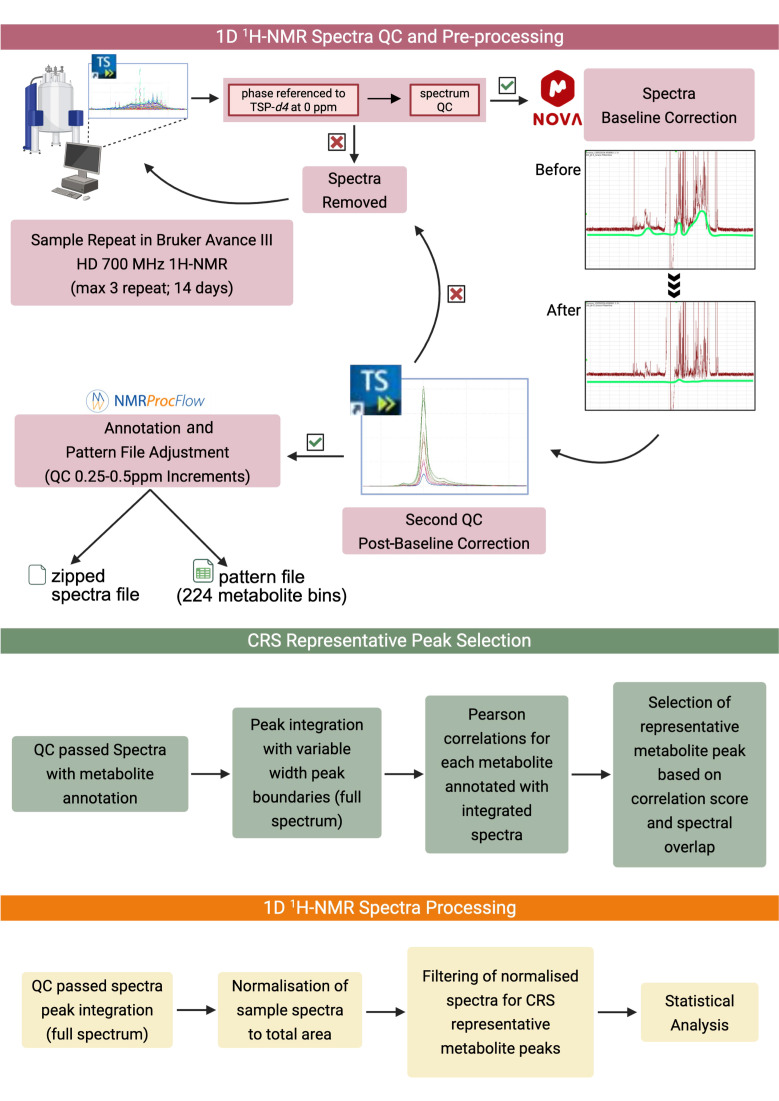
NMR spectral analysis pipeline. The figure above illustrates the 1D 1H-NMR spectra pre-processing, utilising several metabolomic platforms (TopSpin v4.1.3, MNova v15.1, tameNMR, and NMR Procflow v1.4) followed by a CRS-representative peak-selection process and, finally, a spectra binning process with the CRS-selected bin. Green tick passing to next step in the protocol. Red cross indicates fail and return to previous step in the protocol.

A reliable and collaborative spectra pre-processing workflow across validated metabolomic software tools was employed to support the reproducibility of the data (Figure 1) [18,20,21,22]. Following spectral acquisition and preliminary quality control in TopSpin v4.4.1, the highest-quality spectra from each sample underwent a baseline correction using MestreNova (MNova) v15.1 (Mestrelab Research S.L., Santiago de Compostela, Spain) [23,24], accessed through nmrbox (nmrbox.nmrhub.org) [25], to address baseline distortions around the residual water signal region (4.40–5.00 **δ** ppm), as observed in the spectrum with a weak signal due to a lower sample quantity [18,26]. MNova v15.1 provided semi-automated baseline-correcting algorithms [27,28,29] using a Bernstein polynomial approach [30], as demonstrated in previous metabonomic studies [31,32]. By employing this method, the polynomial shapes were manually adjusted using between 10 and 20 inflection points, defined along the baseline per spectrum to fit the undulations and distortions in the baseline prior to the auto adjustment. The corrected spectra from MNova v15.1 were exported in the standardised NMR spectra format ‘Joint Committee on Atomic and Molecular Physical’ (JCAMP-DX; .jdx) prior to the data import into the Bruker proprietary format (1r) and a further secondary spectrum quality check in TopSpin v4.4.1. Any remaining baseline distortions or subsequent baseline corrections that did not meet the quality standards were removed and reprocessed in MNova v15.1 until the quality standards were passed. Once quality-checked, all the spectra that passed QC in TopSpin v4.4.1 were uploaded into NMR Procflow v1.4 [33] for a precise bin adjustment (ppm threshold) and further metabolite annotation.

### 2.6. Annotation for Neutrophil Intracellular Metabolomic Profiling Using CRS-Selected Bins

Following the established guidelines and practice recommendations, the manual annotation of neutrophil metabolites was performed using in-house tools (tameNMR; https://github.com/PGB-LIV/tameNMR, accessed on 1 June 2024) and the profiling software NMR Procflow v1.4 (https://nmrprocflow.org/) [33]. Metabolite peaks were then compared against several open-access metabolite databases, including the HMDB (https://hmdb.ca, accessed on 1 July 2024) [4], Biological Magnetic Resonance dataBank (BMRB; bmrb.io) [34], Chenomx library (ChenomX NMR Suite v8.2), in-house reference library, and prior publications on intracellular neutrophil metabolomics. Then, the annotation accuracy and consistency were ensured through guidance from an expert spectroscopist. Moreover, the biological context was considered crucial when assigning identities to previously unidentified peaks.

A spectral binning process, defining left and right boundaries for each peak multiplet, was employed to select and integrate peaks and peak multiplets while accommodating minimal chemical misalignments of the computed area under the peak in each aligned spectrum. Where possible, the peaks were annotated to known metabolites or marked as unknown. A representative peak bin for each annotated metabolite was selected via correlation reliability score (‘CRS’) filtering [35] through a comparison of the Pearson correlation matrices using R v4.2. This approach yielded a single representative bucket for each annotated metabolite, reducing the number of variables, but also removing unknown NMR peaks. As such, the analysis of the fully integrated spectra (containing information on the known and unknown metabolite peaks), as well as the filtered spectra, containing only representative peaks from all the annotated metabolites present, were both compared in a subsequent spectral analysis.

### 2.7. Data Availability

All the data acquired, processing steps, parameters, metabolite annotations, and CRS representative peak selections were deposited in the European Bioinformatics Institute (EBI) open repository for metabolomics (www.ebi.ac.uk/metabolights) with the ID MTBLS6220 [36].

### 2.8. Statistical Analysis

All the data pre-processing and statistical analyses were performed in R v4.2.0, including supervised and unsupervised multivariate analyses using the mixOmics package [37]. The experimental data were pre-processed using total area (TA) normalisation and tested for normality with the Shapiro–Wilk test prior to the statistical analyses. A univariate analysis was carried out using an ANOVA with Tukey’s post hoc analysis for comparisons among more than two groups, adjusting for multiple comparisons to control the family-wise error rate, and Student’s *t*-test for two-group comparisons, with the application of an adjusted *p*-value of <0.05 using the Benjamini–Hochberg (BH) false discovery rate (FDR) method. For multivariate analyses, the normalised data were Pareto-scaled before applying a PCA and a partial least squares discriminant analysis (PLS-DA). The PLS-DA model was built upon a random selection with a 70:30 (training/test) ratio using 10 randomly sampled model replicates, each with a leave-one-out cross-validation (LOOCV) [38,39,40,41,42]. Besides the mixOmics package mentioned earlier, the automatic iterations of predictive modelling and cross-validations were performed with the caret package in R v4.2.0 [37,43]. The model performance was evaluated using key metrics—the accuracy, balanced accuracy, sensitivity, specificity, precision, and F1 score—of each iteration by directly specifying different numbers of components to evaluate the best number of components needed to build the predictive model [44]. The balanced error rates (BERs) were also calculated for every component by projecting test samples onto latent variables and measuring their Mahalanobis distances from the training data. This calculation incorporates correlations among components to enhance the model robustness, minimise overfitting, and maximise the predictive accuracy. Each metric was calculated individually across each model replicate to determine the model robustness with different training samples. The mean values for each performance metric, along with their respective standard deviation (SD), were then computed to provide a summary of the model reliability and consistency. These averaged metrics offer insight into both the overall predictive power and the stability of the model. Lastly, to assess the contribution of each metabolite to the predictive model, the variable importance in projection (VIP) scores were derived for each model.

## 3. Results

### 3.1. Spectral Binning and Metabolite Annotation

In this study, the challenge was to optimise the NMR protocols for use with clinical samples from paediatrics, where ethical approvals restricted the collection of only 2–3 mL of peripheral whole blood. This typically results in the isolation of less than 10^6^ neutrophils. To address this challenge, we developed an optimisation strategy by designing a stepwise concentration protocol, targeting total cell counts of 100,000, 200,000, 400,000, 800,000, 1.6 × 10^6^, 3.2 × 10^6^, and 5 × 10^6^ neutrophils. Following similar approaches in our published protocols, the number of scans (NS) was varied from the previously established 256 scans to higher NS values of 512, 1024, and the maximum of 2048 [13]. A strategy involving an increased number of scans (NS), or transients, was employed to compensate for the lower metabolite concentration in the extracted neutrophil pellet. Increasing the number of scans (also referred to as transients) in the NMR experiments enhanced the signal intensity as the signal amplitudes were added together, effectively reducing the noise associated with low cell counts. However, this approach also extended the overall acquisition duration. Furthermore, doubling the number of scans only led to a root-square-of-2 improvement in the signal-to-noise ratio, thus limiting the signal improvement gains as the acquisition durations lengthened. In addition, the large dynamic range of signals from the relatively high-concentration residual ^1^H_2_O signal to the low-concentration metabolite peaks increased the baseline distortion, necessitating manual phasing with a baseline correction in addition to the routine automated phasing and baseline correction macro (apk0.noe). Consequently, this allowed for more reliable metabolite quantification, even for low-concentration metabolites like NAD and NADP, which often present weak spectral peaks in highly diluted samples, such as those with a low amount of input material. We also optimised the neutrophil isolation protocol by comparing an ultra-pure, antibody-based negative selection isolation protocol with a density-centrifugation protocol. The negative selection protocols typically used 40–50% fewer neutrophils from the same amount of peripheral blood [45], so it was important to establish any differences in the metabolite profile of neutrophils isolated using both protocols to determine any advantage of ultra-pure methods over the lower yield of cells.

The extracted metabolite spectra from each of the neutrophil cell preparations were acquired using the CPMG experiment, with four different NSs: 256, 512, 1024, and 2048. The spectra were separated into 224 distinct, variable-sized bins. Following CRS filtering, 55 bins were confidently selected to represent 57 annotated metabolites (Supplementary Table S1), while 94 bins corresponding to unknown metabolite peaks were removed. The full spectra (inclusive of unknown metabolites) were also analysed using the same workflow.

### 3.2. Comparing Different Isolation Techniques for Samples

**Figure 2:**
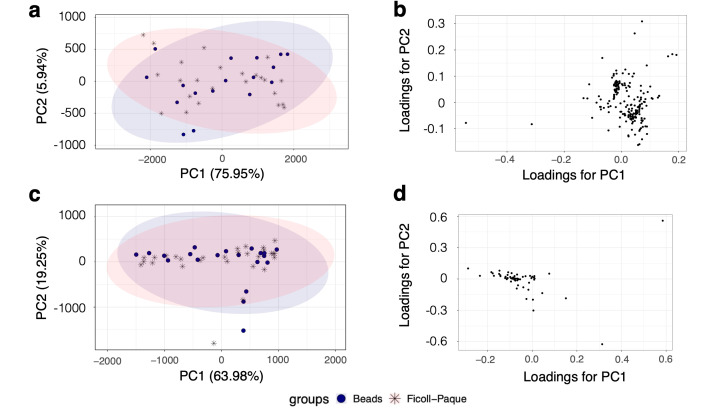
PCA of pre- and post-CRS filtering on optimisation of neutrophil sample spectra based on isolation methods. Pre-CRS filtering: (**a**) PCA score plot and (**b**) PCA loading plot. Post-CRS filtering: (**c**) PCA score plot and (**d**) PCA loading plot. Neutrophils were isolated using Ficoll-Paque (*n* = 28, pink asterisk) or antibody-based negative selection using magnetic beads (*n* = 21, blue circle).

A comparison of NMR metabolite profiles via an unsupervised PCA score plot (Figure 2a) and loading plot (Figure 2b) addressed the gaps in the knowledge about the effect of different neutrophil isolation techniques on intracellular metabolite profiles. The comparison showed a unified clustering for both isolation methods, antibody-based negative selection using magnetic beads (*n* = 21), and density-gradient isolation using Ficoll-Paque (*n* = 28). This indicates that the metabolic profiles from both methods shared similar variance structures, demonstrating that both isolation protocols yielded consistent and comparable results. Such clustering indicates minimal methodological bias and reinforces the reliability of these isolation approaches for further metabolomic analyses. CRS filtering reduced the variance in the data (Figure 2c,d), and the two different isolation techniques could not be distinguished from the post-CRS PCA score plot.

### 3.3. Effect of Cell Count on Metabolite Profile

**Figure 3:**
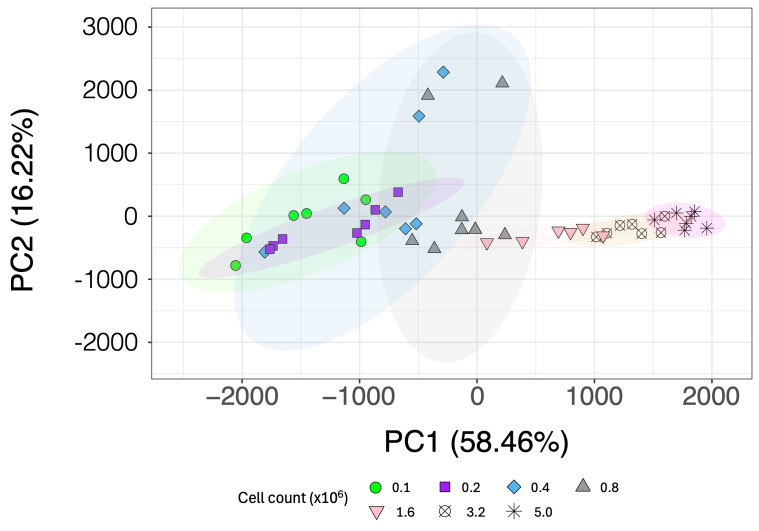
PCA score plot of adult healthy control neutrophils (*n* = 7–8), each with seven different titrations of the cell number (100,000, 200,000, 400,000, 800,000, 1.6 × 10^6^, 3.2 × 10^6^, and 5 × 10^6^) isolated with antibody-based negative selection using magnetic beads or Ficoll-Paque Plus at an NS of 256.

To address the question of whether different neutrophil cell counts would introduce biases due to a decreasing signal-to-noise ratio (S/N) and increased noise contributions in the peaks from low-concentration metabolites, another unsupervised multivariate analysis using a PCA was conducted. The PCA score plot shown in Figure 3 illustrates data from various cell counts: 100,000, 200,000, 400,000, 800,000, 1.6 × 10^6^, 3.2 × 10^6^, and 5 × 10^6^ neutrophils (each *n* = 7–8). The distribution of samples in PC1 and PC2 indicates that greater variance is exhibited in cell counts below 1,600,000 neutrophils.

### 3.4. Comparing the Effect of Increasing the Number of Scans on Low-Cell-Count Neutrophil NMR Metabolic Profiles

**Figure 4:**
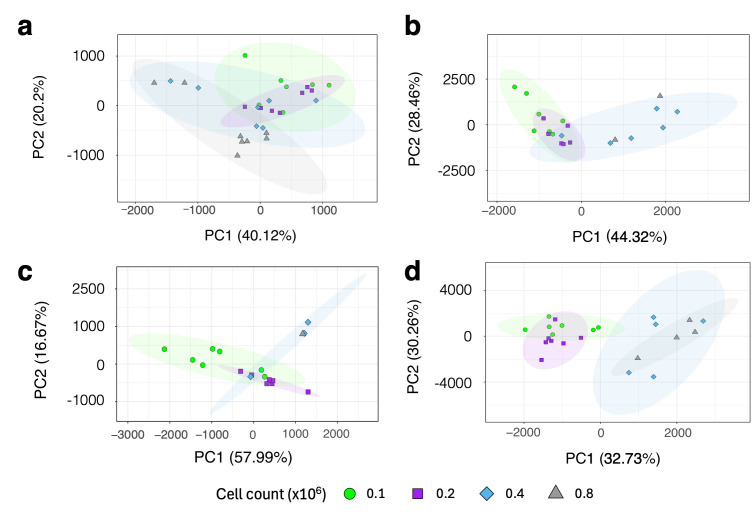
Effect of low cell number on neutrophil metabolomics. PCA score plot of adult healthy control neutrophils (*n* = 7) with 4 different titrations of cell number (100,000, 200,000, 400,000, and 800,000) isolated with antibody-based negative selection using magnetic beads and Ficoll-Paque Plus analysed with increasing scan numbers: (**a**) NS of 256, (**b**) NS of 512, (**c**) NS of 1024, and (**d**) NS of 2048.

Building on the observations from Figure 3, we explored whether increasing the NS would enhance the data quality, particularly for samples containing neutrophil counts of 800,000 cells or fewer. To investigate this, a progressive series of varied scan experiments was conducted, starting from the standard 256 (Figure 4a) and incrementally doubling to 512 (Figure 4b), 1024 (Figure 4c), and 2048 (Figure 4d). The results demonstrated that gradually increasing the NS led to a clearer data structure, with the 2048 scan acquisitions exhibiting distinct clustering between the lowest cell counts (100,000 and 200,000) compared to the higher cell counts of 400,000 and 800,000 (each *n* = 7).

**Figure 5:**
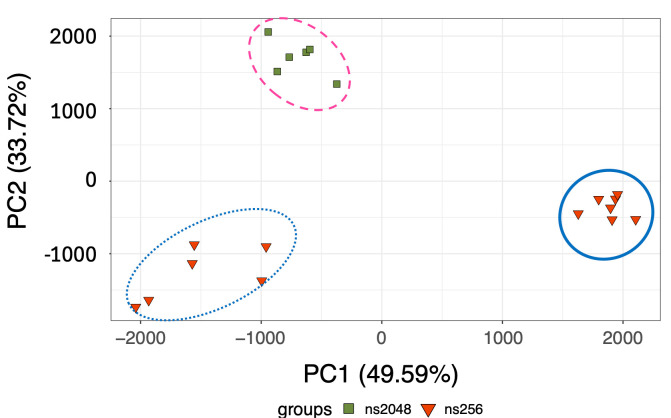
PCA score plot of adult healthy control neutrophils, each with two extreme cell counts (100,000 vs. 5 × 10^6^) from two different NSs, a standard NS of 256 and the highest NS of 2048. The blue dotted line (bottom left) highlights the cluster of samples with 100,000 cells scanned at an NS of 256 (*n* = 6), while the dashed pink line (top) shows the cluster of the same samples scanned at the higher NS of 2048. Additionally, the solid blue line (right) indicates samples with the standard concentration of 5 × 10^6^ cells scanned at the typical NS of 256 (*n* = 7).

Lastly, we wished to confirm whether the lowest cell counts may benefit from using the maximum scan number, an NS of 2048. The PCA suggested that the trend was consistent when applying an unsupervised multivariate analysis with a PCA to assess the clustering patterns by combining samples with the lowest (100,000 cells, *n* = 6) and highest (5 × 10^6^, *n* = 7) cell counts and comparing the standard (NS of 256) and highest (NS of 2048) scan numbers. As shown in Figure 5, the clustering of neutrophils scanned using the standard NS of 256 was influenced by different cell counts, with the lowest cell count exhibiting loose clustering (bottom left). Notably, the 5 × 10^6^ cell samples formed a tighter cluster on the right side of the PCA plot compared to the lowest cell counts (100,000) at an NS of 256. However, when the same low-cell-count sample was scanned with a much higher scan number (NS of 2048), the samples with 100,000 cells exhibited an improved clustering, albeit remaining distinctly separate from the standard cell count samples (5 × 10^6^ cells).

### 3.5. Comparison of Neutrophil Isolation Techniques Using Supervised Multivariate Analysis by PLS-DA

**Figure 6:**
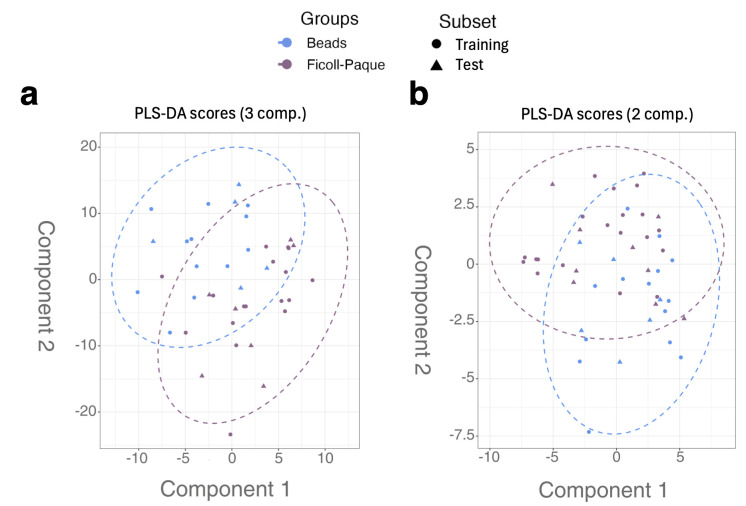
PLS-DA results of pre- and post-CRS filtering on optimisation of neutrophil sample spectra. PLS-DA score plots: (**a**) pre-CRS filtering and (**b**) post-CRS filtering, based on isolation methods (antibody-based negative selection using magnetic beads (*n* = 21, blue circles) or Ficoll-Paque (*n* = 28, purple triangles)).

**Figure 7:**
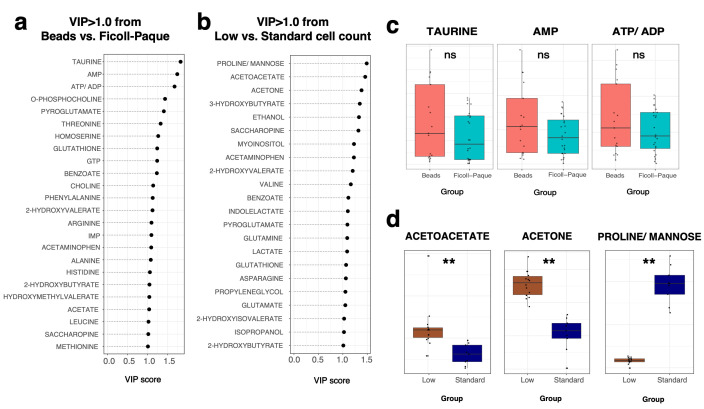
Ranked metabolite bins with VIP scores greater than 1 identified during the optimisation phase for low-count neutrophil samples. Metabolites shown based on (**a**) isolation method (beads, *n* = 21; Ficoll-Paque, *n* = 28) and (**b**) comparison between low (*n* = 14) and standard (*n* = 9) cell counts at NS of 2048. Boxplots show normalised abundance of most influential metabolites for classifying (**c**) isolation protocol and (**d**) cell count (** adj. *p*-value < 0.01, ns = not significant).

A supervised multivariate analysis was performed using a PLS-DA to further explore the discriminatory performance across varying cell counts and neutrophil isolation techniques following CRS filtering. The dataset was divided randomly into 70% training and 30% testing sets for model evaluation, with the predictive performance assessed using accuracy metrics. The PLS-DA score plots in Figure 6 illustrate the distribution of metabolomic data across different neutrophil isolation techniques. Models were built for both the entire spectra (Figure 6a, pre-CRS filtering) and post-CRS filtering (Figure 6b). The corresponding average prediction model performance metrics were calculated from 10 individual randomly sampled model iterations and are shown in Table 1. Using a supervised analysis with a PLS-DA, both the entire spectra and the post-CRS filtering datasets revealed a marginal, yet more discernible, clustering between the samples isolated using magnetic bead isolation (*n* = 21) vs. Ficoll-Paque Plus (*n* = 28). Notably, the pre-CRS filtering model utilised three components, whereas the post-CRS filtering model required only two components, highlighting the refinement in the spectral data following CRS filtering. The PLS-DA model identified 25 significant metabolites with a VIP score > 1 that contributed to differentiating the magnetic bead vs. Ficoll-Paque Plus cell isolation methods (Figure 7a, left). Key metabolites such as taurine, AMP, and ATP/ADP (overlapped) showed a higher abundance and dispersion in magnetic-bead-processed samples compared to Ficoll-Paque (Figure 7a). The visualisation of these metabolites by boxplots showed that neutrophils isolated by Ficoll-Paque had a more homogeneous metabolite profile compared to the bead isolation method (Figure 7c). While the metabolites had a VIP score > 1 in the PLS-DA model, none of these were significantly different between the groups according to the univariate test (adj. *p*-value > 0.05).

The evaluation of the spectra based on varying cell counts across different scans demonstrated the model’s ability to robustly discriminate between different cell concentrations. Firstly, low (100,000 and 200,000, *n* = 14) vs. standard cell counts (400,000 and 800,000, *n* = 9) were compared across different scan numbers (NS of 256 and NS of 2048) (Table 1) and between the full and CRS-filtered datasets. At an NS of 256, the noise was high, and as such, the variance between differing cell counts was not as clearly defined. Using the 2048 NS experiment, the PLS-DA model could clearly discriminate between 100 + 200 and 400 + 800 with a 100% accuracy. A total of 23 metabolites were identified with a VIP > 1 in the PLS-DA model scanned at an NS of 2048 (Figure 7b), where, initially, 20 metabolites were detected through the univariate analysis, demonstrating a high degree of overlap, yet reaffirming the added value of multivariate approaches in metabolomic profiling. Key metabolites such as proline/mannose (overlapped bin), acetoacetate, and acetone exhibited different abundance patterns between the two groups and were significantly different according to the univariate test (adj. *p*-value < 0.01), as shown in the box plots (Figure 7d). Importantly, proline/mannose was only detected at extremely low levels in lower cell counts, suggesting that this metabolite cannot be accurately measured when the cell counts fall below 400,000. Supplementary Figure S2 demonstrates the increased detection of ATP in the NMR spectra with an increasing total cell count (100,000–800,000 cells).

Altogether, these analyses lead us to conclude that a minimum cell count of 400,000 neutrophils is required to measure metabolite signals above the background noise at a standard NS of 256, and that increasing the NS to 2048 does not significantly enhance metabolite detection in samples with cell counts below 400,000. This raises the possibility of obtaining semi-quantitative data normalised to the cell number and comparable between cell numbers of 400,000 or more. However, below this value, increasing the number of scans cannot recover a sufficient signal-to-noise ratio. Based on our experience, it should be possible to obtain 400,000 neutrophils from 5 mL of whole blood from a healthy adult using the Ficoll-Paque isolation protocol presented.

## 4. Discussion

NMR spectroscopy, particularly 1D ^1^H-NMR, has proven to be an invaluable tool in human metabolomics, offering comprehensive analyses of the metabolic profiles of various biological systems and providing critical insight into disease perspectives and patient prognoses. In this study, we applied NMR spectroscopy to optimise sample handling and the refinement of spectra and data pre-processing for low-input cellular material, an area that remains poorly understood. One advantage of our approach is that our protocol for the preparation of extracts used for 1D ^1^H-NMR metabolomics retained the volatile and semi-volatile sample components post-freeze-drying (lyophilisation). This has been observed in numerous previous studies and disciplines, a fact that makes cell extracts an attractive analytical method for the investigation of human disease pathogenesis. However, while metabolomics has seen significant advancements in a variety of human samples, neutrophil-specific studies remain sparse.

To address the challenges associated with low-yield neutrophil samples, particularly those collected from paediatric patients or people with disease-induced neutropenia, where peripheral whole-blood samples often yield fewer than 10^6^ inactivated neutrophils, this study sought to fill a critical methodological gap. A previous study demonstrated the preparation and analysis of neutrophils with as few as 2.5 × 10^6^ cells [13]. However, the limitations posed by paediatric blood samples results in significantly lower neutrophil counts in single blood samples, often falling far below the smallest cell counts analysed in earlier studies. The low cell count inherent to such samples presents significant obstacles for metabolomic profiling, including a reduced signal intensity and increased noise in the NMR spectra, complicating metabolite annotation and downstream analyses. To overcome these limitations, we developed a stepwise concentration protocol tailored to accommodate varying neutrophil counts. This approach systematically targeted cell counts at 100,000, 200,000, 400,000, 800,000, 1.6 × 10^6^, 3.2 × 10^6^, and 5 × 10^6^ cells, ensuring scalability across diverse sample yields. This design provides a robust framework for metabolomic investigations involving low-yield neutrophil samples, establishing a foundation for more reliable and reproducible metabolite detection, even under challenging and suboptimal experimental conditions derived from real clinical samples. This was demonstrated in the results of the PCA clusters for higher cell counts (e.g., 3.2 × 10^6^ or 5 × 10^6^ cells) appearing to be more tightly grouped, suggesting improved data quality and minimal noise. In contrast, clusters from lower cell counts (100,000 and 200,000) displayed more scattering, possibly indicating variability due to noise and a reduced S/N. This dispersion underscores the challenges associated with analysing low-count samples where weaker signals can challenge the analysis, leading to an increased variability and potential biases in metabolite identification and quantification. The VIP analysis identified several metabolites as key discriminators between different cell counts, including proline/mannose, acetoacetate, acetone, lactate, and acetaminophen. Many of these metabolites were significantly different between the lowest and highest cell counts, indicating that the cell number is the most important parameter for capturing accurate metabolic data from human neutrophils.

When we challenged our experiment with a higher number of scans, markedly with the highest NS of 2048, the samples depicted distinct clustering of the lowest cell counts (100,000 and 200,000) compared to the higher cell counts of 400,000 and 800,000, indicating an improved signal quality and data differentiation. Notably, while the signal improved with higher scan numbers, a practical limit was observed; signals from samples with fewer than 400,000 cells (e.g., 100,000) did not consistently overlap with those from higher cell counts (≥400,000), indicating a threshold below which reliable recovery becomes challenging. Collectively, these findings support the idea that increasing the scan number to 2,048 results in reducing the noise interference. This improved the clustering of samples, even for those with the lowest cell counts, thereby corroborating earlier observations [13]. This indicates that a higher scan number can yield more consistent and reliable data, especially for low-abundance samples. A practical limitation remains for samples with fewer than 400,000 cells, which result in several metabolite losses. These findings support a balance between sample availability and analytical robustness, facilitating metabolomic investigations in a challenging clinical sampling situation.

Additionally, this study compared neutrophil isolation methods: magnetic beads vs. Ficoll-Paque. Subtle clustering in the unsupervised multivariate analysis prior to CRS filtering suggested the importance of the CRS-filtering process. Some discrepancies may still arise, despite our work in achieving a neutrophil purity (>95%) comparable to that reported in other studies [13,45,46,47,48] when isolating neutrophils from human peripheral whole blood using magnetic beads and Ficoll-Paque. While no metabolomic studies have specifically explored the influence of various neutrophil isolation methods, prior research has assessed their impact on transcriptomic analyses. For example, a published study comparing density-gradient isolation using negative selection with magnetic beads demonstrated that the primary contaminants identified were low-yield eosinophils, similar to our study [45]. Eosinophil-associated transcripts, such as CD9, were detected, but only in minimal quantities. Despite achieving ultrapure neutrophil populations, the negative selection method also showed trace levels of contaminating cell transcripts. Importantly, these contaminants did not influence the cytokine treatment responses in vitro, indicating that the minor contamination did not compromise the experimental outcomes and both isolation methods were deemed adequate for neutrophil preparation from whole blood and reliable for further downstream applications. Our metabolomic analysis of neutrophils demonstrated that, while both isolation techniques delivered an equivalent quantity of metabolites, with no significant differences in the metabolite abundances between isolation methods, untargeted metabolomics using ^1^H-NMR was sensitive to the relative abundances of these metabolites following different neutrophil isolation methods, highlighting the importance of consistent protocols in neutrophil metabolomic research.

When analysing the spectra from the low-cell-number samples, we applied a number of computational methods to improve the baseline correction and increase the signal-to-noise ratio. Although several types of proprietary software are available for automated baseline corrections in 1D NMR, such as MestreNova, NMR Suite, TopSpin, and KnowItAll [27,28,29], this study utilised Mnova for its advanced capabilities, efficiency, and user-friendly interface. Prior studies have demonstrated that a baseline correction is a critical step in spectral processing, ensuring that distorted or drifting baselines do not compromise the accuracy of metabolite identification [31,32]. The Mnova algorithm initially identifies key points on baseline regions across the spectrum where drift or noise is present, which then can be readjusted to minimise distortions and achieve a better alignment before applying the automated Bernstein polynomial correction [30] algorithm to smooth the baseline and ensure consistent baseline corrections across the spectrum. This automated approach is particularly advantageous because it reduces user-induced variability and improves reproducibility. Moreover, the Mnova functionality effectively handles common challenges in NMR data processing by mitigating water peak distortion, such as that encountered in this research, without additional pre-processing steps.

Our study also built upon the foundational study by Chokesuwattanaskul et al. [13], and successfully identified 57 metabolites distributed across 55 buckets (24.6%) from the initial pool of 224 buckets using an intensive annotation workflow involving NMR Procflow (v1.4), ChenomX NMR Suite (v8.2), and TopSpin (v4.4.1). This achievement highlights progress in untangling the complexities of neutrophil metabolomic analyses. However, challenges persist due to the inherently small chemical shift window of 1D ^1^H-NMR, which often leads to overlapping resonances, particularly in the 1–4 ppm region [1,49,50]. This region is densely populated by biofluid metabolites, and is further compounded by interference from spectral overlaps between sample metabolites in NMR experiments. To address these challenges, our study employed a refined metabolite annotation strategy aimed at reducing multicollinearity within the spectral data, leveraging CRS scoring to account for metabolite correlations, and incorporating the biological context to enhance the reliability and interpretability [35,51]. As a result, while most metabolites were confidently assigned to their respective CRS-refined bins, two bins remained ambiguous due to overlapping signals from multiple metabolites, with mannose and proline sharing the same bin as well as both ATP and ADP.

While the experiments detailed here have improved our understanding of the effect of neutrophil isolation methods and the limits of the cell counts tractable by NMR, it is important to stress the need for consistent methods of isolation, although we believe that both methods shown are equivalently suited to NMR metabolomics in terms of metabolite coverage and the variance of the metabolome. However, our analysis was limited by the small sample size, constraining the validation method to LOOCV alone rather than more robust methods like bootstrapping or advanced machine learning techniques. This limitation underscores the inherent risk of overfitting in highly multicollinear metabolomic datasets. CRS refinement has somewhat mitigated the limits of small sample sizes, enhancing the dataset quality by selecting representative metabolite bins to effectively reduce noise and facilitate a more robust differentiation of metabolite profiles through clearer clustering and distinctions among groups in multivariate analyses. However, the untargeted approach comes with significant challenges, particularly in the identification and assignments of unknown metabolites. Annotating both recognised and unknown metabolites from NMR spectra remains a challenge. As proposed by Sumner et al. [17], the confidence levels for metabolite annotations in published metabolomic studies are classified into four of the Metabolomics Standards Initiative (MSI) categories: (1) identified compounds—non-novel compounds with a confirmed name, structure, CAS number, or international chemical identifier (InChI); (2) putatively annotated compounds with identified physicochemical properties/spectral similarity without known chemical reference standards; (3) putatively identified compounds using their compound classes; and (4) unknown compounds. To date, manual untargeted metabolite annotation pipelines remain complex and laborious, arising from a small chemical shift window in 1D ^1^H-NMR. This leads to overlapping resonances, particularly in the region between 1 and 4 ppm, where most metabolites present in biofluid samples arise (aliphatic molecules such as saccharides, amino acids, etc.) [1,22,49,50]. Other possible challenges in metabolite annotation, including chemical shift differences, can arise from the effect of the ionic strength of the samples, the sample pH, the chemical interaction of metabolites within the samples, and the solvent [49,50,52]. Adherence to best practice ensures the reproducibility and reliability of the neutrophil metabolite spectra prior to a data analysis. This includes sample handling and processing using HEPES-free media, the use of pH buffers and an internal chemical shift reference to TSP-d4, good water suppression, and shimming, as well as the optimisation of the ^1^H nuclear Overhauser effect spectroscopy (NOESY) experiment and the subsequent quality control (QC) measures, which were carried out on each sample.

## 5. Conclusions

In conclusion, we optimised an NMR analysis pipeline for a metabolomic analysis of human neutrophils with a low amount of input material. We demonstrated that the minimum cell count required for the accurate detection of neutrophil metabolites at a standard NS of 256 is 400,000 cells. Spectral processing and a baseline correction using Mnova improved the signal-to-noise ratio of the spectra from samples with low cell counts. The application of CRS filtering to the pattern file preserved the essential discriminatory features of our PLS-DA models and improved the dataset robustness and analytical precision. In addition, we demonstrated that both neutrophil isolation protocols investigated, centrifugation through Ficoll-Paque and negative selection using antibody-based magnetic bead protocols, are equivalently suited for NMR metabolomics. We believe that our optimised protocol provides a method for NMR metabolomic analyses of samples with low input cell counts, which can be adopted into translational multiomics studies of paediatrics and rare diseases where access to material is limited.

## Figures and Tables

**Table 1:** PLS-DA prediction model metrics presented in means ± standard deviation.

Dataset	Bin	Components	Accuracy	Sensitivity	Specificity	Precision	F1 Score
**Magnetic beads vs.** **Ficoll-Paque**	**Original**	3	0.87 ± 0.11	0.89 ± 0.12	0.82 ± 0.15	0.88 ± 0.10	0.88 ± 0.09
	**Post-CRS**	2	0.74 ± 0.12	0.83 ± 0.15	0.64 ± 0.19	0.74 ± 0.14	0.78 ± 0.12
**NS 256 ** **100 + 200 (low) vs. 400 + 800 (standard) ** **cell count**	**Original**	1	0.82 ± 0.12	0.86 ± 0.16	0.77 ± 0.21	0.81 ± 0.19	0.82 ± 0.14
	**Post-CRS**	1	0.85 ± 0.09	0.93 ± 0.13	0.79 ± 0.19	0.83 ± 0.17	0.86 ± 0.09
**NS 2048 ** **100 + 200 (low) vs. 400 + 800 (standard) ** **cell count**	**Original**	2	1.00 ± 0.00	1.00 ± 0.00	1.00 ± 0.00	1.00 ± 0.00	1.00 ± 0.00
	**Post-CRS**	2	1.00 ± 0.00	1.00 ± 0.00	1.00 ± 0.00	1.00 ± 0.00	1.00 ± 0.00

## Data Availability

All the data acquired, processing steps, parameters, metabolite annotations, and CRS representative peak selections were deposited in the European Bioinformatics Institute (EBI) open repository for metabolomics (www.ebi.ac.uk/metabolights) [36].

## Supplementary Materials

The following supporting information can be downloaded at: https://www.mdpi.com/article/10.3390/metabo15090612/s1, Figure S1: Comparison of spectra acquired by CPMG (purple) and NOESY (orange) methods. The 1–3 ppm region shows a minor background from macromolecules present in the neutrophil extracts acquired by the NOESY experiment; Figure S2: Spectra showing ATP (8.3 ppm and 8.55 ppm) visibility with 100, 200, 400, and 800 thousand neutrophils (bottom to top) at an NS of 256. The figure was plotted using CCPN (https://ccpn.ac.uk) [53] and the CASMDB metabolite standards library [54] ; Table S1: The table of annotated metabolites from human neutrophil spectra, with the HMDB ID, MSI confidence level, and number of representative bins.

## Abbreviations

The following abbreviations are used in this manuscript:

ADP	adenosine diphosphate
AMP	adenosine monophosphate
ATP	adenosine triphosphate
COMETS	Consortium of Metabolomics Studies
CPMG	Carr–Purcell–Meiboom–Gill
CRS	correlation reliability score
FDR	false discovery rate
LOOCV	leave-one-out cross-validation
NAD	nicotinamide adenine dinucleotide
NADP	nicotinamide adenine dinucleotide phosphate
NMR	nuclear magnetic resonance
NOESY	nuclear Overhauser effect spectroscopy
NS	number of scans
PCA	principal component analysis
PLS-DA	partial least squares discriminant analysis
S/N	signal-to-noise ratio
VIP	variable importance in projection

## References

[1] Emwas A.M., Salek R.M., Griffin J.L., Merzaban J. (2013). NMR-based metabolomics in human disease diagnosis: Applications, limitations, and recommendations. Metabolomics.

[2] Amaral P., Carbonell-Sala S., De La Vega F.M., Faial T., Frankish A., Gingeras T., Guigo R., Harrow J.L., Hatzigeorgiou A.G., Johnson R. (2023). The status of the human gene catalogue. Nature.

[3] Wishart D.S. (2019). Metabolomics for Investigating Physiological and Pathophysiological Processes. Physiol. Rev..

[4] Wishart D.S., Guo A., Oler E., Wang F., Anjum A., Peters H., Dizon R., Sayeeda Z., Tian S., Lee B.L. (2022). HMDB 5.0: The Human Metabolome Database for 2022. Nucleic Acids Res..

[5] Hassan M.A., Al-Sakkaf K., Shait Mohammed M.R., Dallol A., Al-Maghrabi J., Aldahlawi A., Ashoor S., Maamra M., Ragoussis J., Wu W. (2020). Integration of Transcriptome and Metabolome Provides Unique Insights to Pathways Associated With Obese Breast Cancer Patients. Front. Oncol..

[6] Kouznetsova V.L., Kim E., Romm E.L., Zhu A., Tsigelny I.F. (2019). Recognition of early and late stages of bladder cancer using metabolites and machine learning. Metabolomics.

[7] Kim W.T., Yun S.J., Yan C., Jeong P., Kim Y.H., Lee I.S., Kang H.W., Park S., Moon S.K., Choi Y.H. (2016). Metabolic Pathway Signatures Associated with Urinary Metabolite Biomarkers Differentiate Bladder Cancer Patients from Healthy Controls. Yonsei Med. J..

[8] Jin X., Yun S.J., Jeong P., Kim I.Y., Kim W.J., Park S. (2014). Diagnosis of bladder cancer and prediction of survival by urinary metabolomics. Oncotarget.

[9] Wright H.L., Moots R.J., Bucknall R.C., Edwards S.W. (2010). Neutrophil function in inflammation and inflammatory diseases. Rheumatology.

[10] Fresneda Alarcon M., McLaren Z., Wright H.L. (2021). Neutrophils in the Pathogenesis of Rheumatoid Arthritis and Systemic Lupus Erythematosus: Same Foe Different M.O. Front. Immunol..

[11] Yu B., Zanetti K.A., Temprosa M., Albanes D., Appel N., Barrera C.B., Ben-Shlomo Y., Boerwinkle E., Casas J.P., Clish C. (2019). The Consortium of Metabolomics Studies (COMETS): Metabolomics in 47 Prospective Cohort Studies. Am. J. Epidemiol..

[12] Richer B.C., Salei N., Laskay T., Seeger K. (2018). Changes in Neutrophil Metabolism upon Activation and Aging. Inflammation.

[13] Chokesuwattanaskul S., Phelan M.M., Edwards S.W., Wright H.L. (2018). A robust intracellular metabolite extraction protocol for human neutrophil metabolic profiling. PLoS ONE.

[14] Chokesuwattanaskul S., Fresneda Alarcon M., Mangalakumaran S., Grosman R., Cross A.L., Chapman E.A., Mason D., Moots R.J., Phelan M.M., Wright H.L. (2022). Metabolic Profiling of Rheumatoid Arthritis Neutrophils Reveals Altered Energy Metabolism That Is Not Affected by JAK Inhibition. Metabolites.

[15] Beckonert O., Keun H.C., Ebbels T.M., Bundy J., Holmes E., Lindon J.C., Nicholson J.K. (2007). Metabolic profiling, metabolomic and metabonomic procedures for NMR spectroscopy of urine, plasma, serum and tissue extracts. Nat. Protoc..

[16] Findeisen M., Brand T., Berger S. (2007). A 1H-NMR thermometer suitable for cryoprobes. Magn. Reson. Chem..

[17] Sumner L.W., Amberg A., Barrett D., Beale M.H., Beger R., Daykin C.A., Fan T.W., Fiehn O., Goodacre R., Griffin J.L. (2007). Proposed minimum reporting standards for chemical analysis Chemical Analysis Working Group (CAWG) Metabolomics Standards Initiative (MSI). Metabolomics.

[18] Ebbels T.M.D., Karaman I., Graca G. (2019). Processing and Analysis of Untargeted Multicohort NMR Data. Methods Mol. Biol..

[19] Kostidis S., Addie R.D., Morreau H., Mayboroda O.A., Giera M. (2017). Quantitative NMR analysis of intra- and extracellular metabolism of mammalian cells: A tutorial. Anal. Chim. Acta.

[20] Vu T.N., Valkenborg D., Smets K., Verwaest K.A., Dommisse R., Lemiere F., Verschoren A., Goethals B., Laukens K. (2011). An integrated workflow for robust alignment and simplified quantitative analysis of NMR spectrometry data. BMC Bioinform..

[21] Powers R., Andersson E.R., Bayless A.L., Brua R.B., Chang M.C., Cheng L.L., Clendinen C.S., Cochran D., Copié V., Cort J.R. (2024). Best practices in NMR metabolomics: Current state. TrAC Trends Anal. Chem..

[22] Huang K., Thomas N., Gooley P.R., Armstrong C.W. (2022). Systematic Review of NMR-Based Metabolomics Practices in Human Disease Research. Metabolites.

[23] Bravo J.A., Vila J., Flores Y. (2018). NMR Mestrenova, short manual for beginners. Boliv. J. Chem..

[24] Forezi L.S.M., Branco F.S.C. (2017). Editing NMR spectra eith MestReNova software: A practical guide. Revisita Virtual De Quim..

[25] Maciejewski M.W., Schuyler A.D., Gryk M.R., Moraru I.I., Romero P.R., Ulrich E.L., Eghbalnia H.R., Livny M., Delaglio F., Hoch J.C. (2017). NMRbox: A Resource for Biomolecular NMR Computation. Biophys. J..

[26] Garcia-Perez I., Posma J.M., Serrano-Contreras J.I., Boulange C.L., Chan Q., Frost G., Stamler J., Elliott P., Lindon J.C., Holmes E. (2020). Identifying unknown metabolites using NMR-based metabolic profiling techniques. Nat. Protoc..

[27] Izquierdo-Garcia J.L., Villa P., Kyriazis A., del Puerto-Nevado L., Perez-Rial S., Rodriguez I., Hernandez N., Ruiz-Cabello J. (2011). Descriptive review of current NMR-based metabolomic data analysis packages. Prog. Nucl. Magn. Reson. Spectrosc..

[28] Sawall M., von Harbou E., Moog A., Behrens R., Schroder H., Simoneau J., Steimers E., Neymeyr K. (2018). Multi-objective optimization for an automated and simultaneous phase and baseline correction of NMR spectral data. J. Magn. Reson..

[29] Wang K.C., Wang S.Y., Kuo C.H., Tseng Y.J. (2013). Distribution-based classification method for baseline correction of metabolomic 1D proton nuclear magnetic resonance spectra. Anal. Chem..

[30] Lorentz (2012). Bernstein Polynomials.

[31] Ralston S.L., Pappalardo L., Pelczer I. (2011). Breed and age effects on metabolic profiles of young horses using NMR-based Metabonomic analyses of serum. J. Equine Vet. Sci..

[32] Corona-Vázquez M., Hernández-Bolio G.I., Muñoz-Cázares N., Peña-González M.C., Derbré S., Richomme P., Peña-Rodríguez L.M. (2024). 1H-NMR-Based Chemometrics and 13C-NMR Dereplication Analysis Applied to the Bioprospecting of the Yucatecan Flora: Identification of 3-O-Acetyl-Ceanotic Acid as an Inhibitor of Bacterial Virulence Factors from Colubrina yucatanensis. Rev. Bras. Farm..

[33] Jacob D., Deborde C., Lefebvre M., Maucourt M., Moing A. (2017). NMRProcFlow: A graphical and interactive tool dedicated to 1D spectra processing for NMR-based metabolomics. Metabolomics.

[34] Hoch J.C., Baskaran K., Burr H., Chin J., Eghbalnia H.R., Fujiwara T., Gryk M.R., Iwata T., Kojima C., Kurisu G. (2023). Biological Magnetic Resonance Data Bank. Nucleic Acids Res..

[35] Grosman R. (2019). NMR Metabolomic Profiling of Mosquito Species to Understand Insecticide Resistance.

[36] Yurekten O., Payne T., Tejera N., Amaladoss F.X., Martin C., Williams M., O’Donovan C. (2024). MetaboLights: Open data repository for metabolomics. Nucleic Acids Res..

[37] Rohart F., Gautier B., Singh A., Le Cao K.A. (2017). mixOmics: An R package for omics feature selection and multiple data integration. PLoS Comput. Biol..

[38] Xu Y., Goodacre R. (2018). On Splitting Training and Validation Set: A Comparative Study of Cross-Validation, Bootstrap and Systematic Sampling for Estimating the Generalization Performance of Supervised Learning. J. Anal. Test..

[39] Rodriguez-Perez R., Fernandez L., Marco S. (2018). Overoptimism in cross-validation when using partial least squares-discriminant analysis for omics data: A systematic study. Anal. Bioanal. Chem..

[40] Gromski P.S., Correa E., Vaughan A.A., Wedge D.C., Turner M.L., Goodacre R. (2014). A comparison of different chemometrics approaches for the robust classification of electronic nose data. Anal. Bioanal. Chem..

[41] Westerhuis J.A., Hoefsloot H.C.J., Smit S., Vis D.J., Smilde A.K., van Velzen E.J.J., van Duijnhoven J.P.M., van Dorsten F.A. (2008). Assessment of PLSDA cross validation. Metabolomics.

[42] Gromski P.S., Muhamadali H., Ellis D.I., Xu Y., Correa E., Turner M.L., Goodacre R. (2015). A tutorial review: Metabolomics and partial least squares-discriminant analysis--a marriage of convenience or a shotgun wedding. Anal. Chim. Acta.

[43] Kuhn M. (2008). Building Predictive Models in R Using the caret Package. J. Stat. Softw..

[44] Szymańska E., Saccenti E., Smilde A.K., Westerhuis J.A. (2012). Double-check: Validation of diagnostic statistics for PLS-DA models in metabolomics studies. Metabolomics.

[45] Thomas H.B., Moots R.J., Edwards S.W., Wright H.L. (2015). Whose Gene Is It Anyway? The Effect of Preparation Purity on Neutrophil Transcriptome Studies. PLoS ONE.

[46] Pelletier M., Maggi L., Micheletti A., Lazzeri E., Tamassia N., Costantini C., Cosmi L., Lunardi C., Annunziato F., Romagnani S. (2010). Evidence for a cross-talk between human neutrophils and Th17 cells. Blood.

[47] Mitchell T.S., Moots R.J., Wright H.L. (2017). Janus kinase inhibitors prevent migration of rheumatoid arthritis neutrophils towards interleukin-8, but do not inhibit priming of the respiratory burst or reactive oxygen species production. Clin. Exp. Immunol..

[48] Wright H.L., Thomas H.B., Moots R.J., Edwards S.W. (2013). RNA-Seq Reveals Activation of Both Common and Cytokine-Specific Pathways following Neutrophil Priming. PLoS ONE.

[49] Emwas A.H., Roy R., McKay R.T., Tenori L., Saccenti E., Gowda G.A.N., Raftery D., Alahmari F., Jaremko L., Jaremko M. (2019). NMR Spectroscopy for Metabolomics Research. Metabolites.

[50] Dona A.C., Kyriakides M., Scott F., Shephard E.A., Varshavi D., Veselkov K., Everett J.R. (2016). A guide to the identification of metabolites in NMR-based metabonomics/metabolomics experiments. Comput. Struct. Biotechnol. J..

[51] Debik J., Sangermani M., Wang F., Madssen T.S., Giskeødegård G.F. (2022). Multivariate analysis of NMR-based metabolomic data. NMR Biomed..

[52] Bharti S.K., Roy R. (2012). Quantitative 1H NMR spectroscopy. TrAC Trends Anal. Chem..

[53]  Skinner S.P., Fogh RH., Boucher W., Ragan T.J., Mureddu L.G., Vuister G.W. (2016). CcpNmr AnalysisAssign: a flexible platform for integrated NMR analysis. J Biomol NMR.

[54] Hayward M.W., Mureddu L.G., Thompson G., Phelan M., Brooksbank E.J., Vuister G.W. (2024). The CcpNmr Analysis Simulated Metabolomics Database (CASMDB): An Open-Source Collection of Metabolite Annotation Data for 1D ^1^H NMR-Based Metabolomics. bioRxiv.

