# Supplementary material for: An NMR Metabolomics Analysis Pipeline for Human Neutrophil Samples with Limited Source Material
Source: Metabolites. 2025 Sep 15;15(9):612. doi: 10.3390/metabo15090612 (PMC12472055; doi:10.3390/metabo15090612)
Supplement: Supplementary file 1 [file metabolites-15-00612-s001.zip › metabolites-3829320-supplementary.pdf]

Supplementary Table S1. The table of annotated metabolites from human neutrophil spectra, with HMDB ID, MSI confidence level, and number of representative bins.

Upper Bound (ppm)	Lower Bound (ppm)	Metabolite	MSI Level	HMDB ID	Number of Representative Bins
2.0094	2.0039	ACETAMIDE	2	HMDB0031645	1
7.1988	7.187	ACETAMINOPHEN	2	HMDB0001859	2
1.9225	1.9169	ACETATE	2	HMDB0000042	1
2.2674	2.2618	ACETOACETATE	2	HMDB0304256	1
2.2385	2.2298	ACETONE	2	HMDB0001659	1
1.57	1.5401	ADIPATE	2	HMDB0040270	3
8.2777	8.2742	AMP	2	HMDB0000045	4
3.8188	3.7872	HOMOSERINE	2	HMDB0000719	12
1.4988	1.4768	ALANINE	2	HMDB0000161	2
3.7834	3.7782	ARGININE	2	HMDB0000517	10
3.2608	3.2541	TAURINE	2	HMDB0000251	3
2.884	2.8424	ASPARAGINE	2	HMDB0000168	3
2.699	2.6676	ASPARTATE	2	HMDB0000191	6
8.5543	8.5407	ADP	2	HMDB0001341	4
8.5543	8.5407	ATP	2	HMDB0000538	7
7.8875	7.8599	BENZOATE	2	HMDB0304270	5
7.5078	7.4824	INDOLELACTATE	2	HMDB0000671	2
3.2129	3.2046	CHOLINE	2	HMDB0000097	1
4.0585	4.0213	CYSTEINE	2	HMDB0000574	1
2.735	2.7313	DIMETHYLAMINE	2	HMDB0000087	1
8.4626	8.4562	FORMATE	2	HMDB0000142	1

Supplementary Table S1 (*cont.*).

Upper Bound (ppm)	Lower Bound (ppm)	Metabolite	MSI Level	HMDB ID	Number of Representative Bins
3.8309	3.823	GLUCOSE	2	HMDB0304632	19
2.3716	2.367	GLUTAMATE	2	HMDB0000148	12
2.1421	2.1379	GLUTAMINE	2	HMDB0000641	6
2.5938	2.5517	GLUTATHIONE	2	HMDB0000125	4
3.6507	3.6424	GLYCEROL	2	HMDB0000131	4
8.1475	8.1429	GTP	2	HMDB0001273	2
7.8365	7.8268	HISTIDINE	2	HMDB0000177	2
1.2572	1.2498	HYDROXYISOVALERATE2	2	HMDB0002011	2
0.872	0.8504	HYDROXYMETHYLVALERATE	2	HMDB0000317	9
1.437	1.38	HYDROXYVALERATE	2	HMDB0001863	3
8.2187	8.2139	IMP	2	HMDB0002271	3
1.0249	1.0054	ISOLEUCINE	2	HMDB0000172	11
1.159	1.1531	ISOPROPANOL	2	HMDB0000863	3
1.3279	1.3224	LACTATE	2	HMDB0001311	3
0.9704	0.9613	LEUCINE	2	HMDB0000687	7
1.767	1.7374	SACCHAROPINE	2	HMDB0000279	17
3.0432	3.0268	LYSINE	2	HMDB0000182	10
3.6294	3.6251	MYOINOSITOL	2	HMDB0000211	6
8.1817	8.1781	NAD	2	HMDB0000902	6
8.1921	8.184	NADP	2	HMDB0000217	4
3.2284	3.2211	OPHOSPHOCHOLINE	2	HMDB0001565	1
7.3786	7.286	PHENYLALANINE	2	HMDB0000159	6
1.0745	1.057	PROPIONATE	2	HMDB0000237	5
1.1531	1.1381	PROPYLENEGLYCOL	2	HMDB0001881	1
2.5303	2.5017	PYROGLUTAMATE	2	HMDB0000267	8
2.5003	2.4262	GLUTAMINE	2	HMDB0000641	6
3.9641	3.9528	SERINE	2	HMDB0000187	4
3.6002	3.579	THREONINE	2	HMDB0000167	3
1.1901	1.183	ETHANOL	2	HMDB0000108	3
1.2241	1.2073	HYDROXYBUTYRATE3	2	HMDB0000011	1
0.9012	0.8962	HYDROXYBUTYRATE2	2	HMDB0000008	1
8.2397	8.2352	NADH	2	HMDB0001487	1
3.365	3.349	MANNOSE	2	HMDB0000169	2
3.365	3.349	PROLINE	2	HMDB0000162	3
2.767	2.7544	SARCOSINE	2	HMDB0000271	1
1.0059	0.9867	VALINE	2	HMDB0000883	6

Supplementary Figure S1. Comparison of spectra acquired by CPMG (purple) and NOESY (orange) methods. The 1-3ppm region shows minor background from macromolecules present in neutrophil extracts acquired by NOESY experiment.

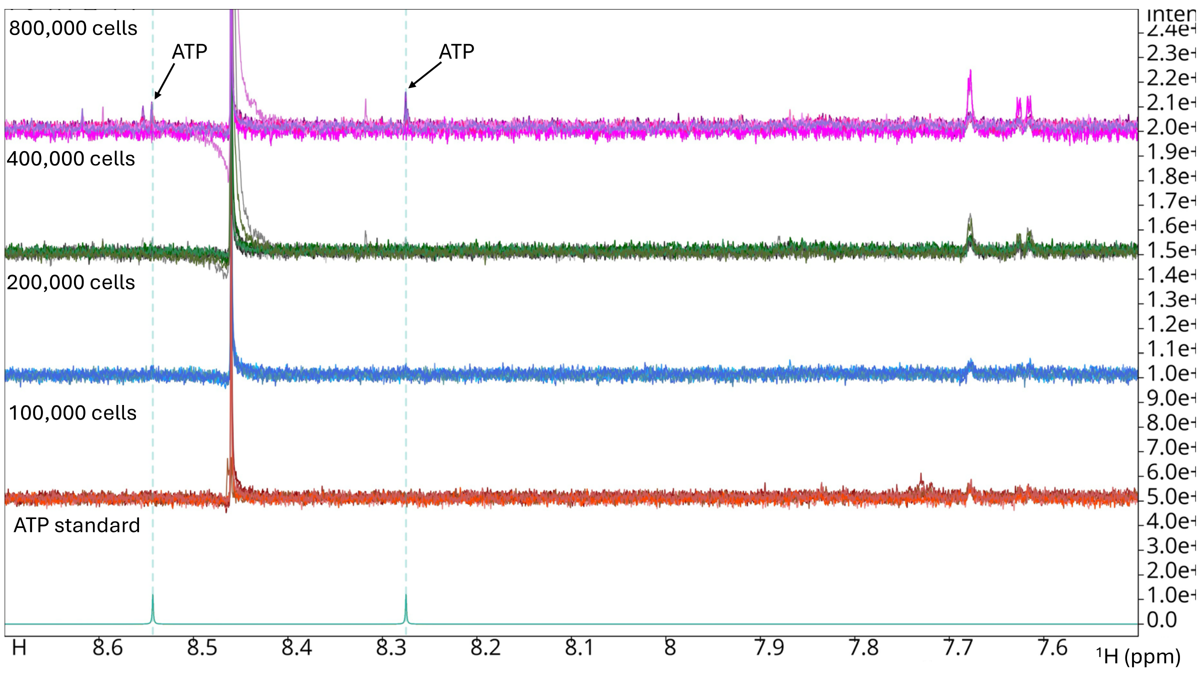

Supplementary Figure S2. Spectra showing ATP (8.3ppm and 8.55ppm) visibility with 100, 200, 400 and 800 thousand neutrophils (bottom to top) at NS 256. Figure was plotted using CCPN (ccpn.ac.uk [53]) and the CASMDB metabolite standards library [54].
